# A Rare Cause of Upper Gastrointestinal Bleeding: Superior Mesenteric Artery Aneurysm

**DOI:** 10.7759/cureus.95311

**Published:** 2025-10-24

**Authors:** Natalia Sinou, Despoina Milonaki, Aristeidis Papadopoulos, Sophia Ispanopoulou, Georgios Meimaris

**Affiliations:** 1 Research and Education Institute in Biomedical Sciences, National and Kapodistrian University of Athens School of Medicine, Athens, GRC; 2 Department of General Surgery, General Hospital of Nikaia "Agios Panteleimon", Athens, GRC; 3 Department of Radiology, General Hospital of Nikaia "Agios Panteleimon", Athens, GRC

**Keywords:** bleeding, gi, pseudoaneurysm, superior mesenteric artery, visceral aneurysm

## Abstract

Superior mesenteric artery (SMA) aneurysms are a rare cause of gastrointestinal (GI) bleeding that is usually underdiagnosed. These conditions can lead to severe complications, making prompt treatment essential.

We report a case of a 87-year-old female patient admitted for intense pain in the abdominal area. After a 24-hour hospital stay, she had an episode of hematochezia, hematemesis, and intense epigastric pain. Upper GI endoscopy revealed a foreign body in the duodenum, which was removed. The bleeding and the hemodynamic instability were persistent even after the removal of the foreign body. A CT angiography revealed an aneurysm in the SMA with active contrast extravasation. The patient underwent endovascular treatment with coil embolization, achieving complete hemodynamic stabilization without complications.

GI bleeding from an aneurysm in the SMA is an unusual but critical entity with high mortality (40-60%); thus, rupture of the SMA aneurysm should always be considered in the differential diagnosis in cases with persistent GI bleeding. Regarding the hemodynamic stability of the patient or the patient’s overall clinical condition, the available treatment options include endoscopic interventions or open surgery. Endovascular treatment offers a safe and effective alternative to open surgery, with favorable outcomes.

Prompt diagnosis and, most of the time, endovascular embolization are crucial for successful management of SMA aneurysm-related upper GI bleeding.

## Introduction

Upper abdominal bleeding can be a serious condition that arises from various causes, including peptic ulcer disease, variceal hemorrhage, vascular malformations, and swallowing foreign objects. Most cases stem from common gastrointestinal (GI) issues, but rare causes like visceral artery aneurysms (VAAs) can also lead to severe GI bleeding. These conditions are often silent and occur in only 0.2% of the general population [1], yet they carry a high risk of rupture and death (25-70%). The superior mesenteric artery (SMA) and its branches are involved in a small number of cases [2], but when a rupture happens, quick recognition and intervention are essential. Foreign body ingestion in older patients, especially those on anticoagulation therapy, presents an added challenge for diagnosis and treatment [3]. In these cases, the clinical presentation may be unusual, which can delay diagnosis and management. Improvements in imaging techniques, like computed tomography (CT) and CT angiography, have made diagnoses more accurate [4,5]. Meanwhile, endovascular methods have become less invasive and very effective treatment options compared to open surgery.

## Case presentation

An 87-year-old female patient presented to the Emergency Department with intense pain in the abdominal area. She reported diffuse abdominal pain for the past 10 days without disturbances in bowel movements, culminating the previous day when the pain was particularly intense. Her vital signs were as follows: blood pressure, 104/73 mm Hg; heart rate, 81 bpm; and oxygen saturation, 97% on room air (FiO₂ 21%). Clinical examination revealed abdominal pain mainly localized in the right iliac fossa, with no other significant findings. She presented hemodynamically and respiratory stable, afebrile, and reports normal bowel movement. The patient is admitted to the Surgical Department of the hospital for further diagnostic investigation and treatment.

Regarding the patient's medical history, she had a spinal fusion surgery, a previous cerebrovascular incident, dyslipidemia, hypertension, hypothyroidism, and organic mental disorder. For this reason, she was receiving anticoagulant and antithrombotic therapy with apixaban and clopidogrel, respectively, atorvastatin, olmesartan medoxomil with hydrochlorothiazide, and duloxetine.

A series of laboratory and radiological examinations was conducted in order to clarify the differential diagnosis. The basic laboratory examination revealed an active inflammatory situation with elevated white blood cells (16.91 × 10⁹/L; 93.5% neutrophils) and C-reactive protein (CRP) (118.8 mg/L), and hemoglobin (Hb), hematocrit (Hct), and platelet (PLT) levels were normal (Hb: 11.6 g/dL, Hct: 35.2, PLT: 388.000 × 10⁹/L) (Table 1). Initial antibiotic treatment with piperacillin-tazobactam and metronidazole was administered.

**Table 1:** Timeline summarizing key clinical events, laboratory changes, and interventions during hospitalization.

Day	Event	Hemoglobin (g/dL)	Hematocrit (%)	WBC (×10⁹/L)	CRP (mg/L)	Intervention
0	Admission	11.6	35.2	16.9	118.8	Antibiotics
1	First bleed	↓	↓	↑	↑	RBC transfusion
2	Endoscopy (foreign body)	-	-	-	-	Clip
5	Second bleed	↓	↓	↑	↑	CTA → embolization
6-10	Recovery	Stable	Stable	↓	↓	-

**Figure 1:**
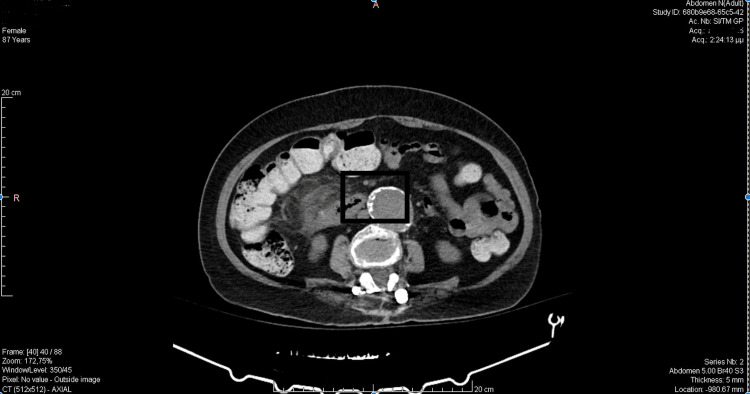
CT scan.

The patient underwent an abdominal computer tomography (CT) scan with intravenous contrast. The CT scan revealed that, adjacent to the cecum, fluid collections and free air bubbles were noted, raising the suspicion of perforation. Regional lymphadenopathy was present. Due to the recent administration of the anticoagulant therapy, as well as the patient’s favorable clinical status, the surgical intervention was postponed (Figure 1).

The abdominal pain continued during the first day of hospitalization (July 2025), and in the evening hours, pethidine was required to alleviate it. It is worth noting that due to the previous spinal fusion surgery, the patient has developed resistance to many analgesics. The intense abdominal pain at night was accompanied by a drop in Hb, so she received her first red blood cell (RBC) transfusion.

However, on the second day of hospitalization, the patient developed acute chest and epigastric pain, accompanied by both upper and lower massive GI bleeding. A cardiology evaluation was performed, which revealed no evidence of acute cardiac pathology. The GI bleeding was managed conservatively with tranexamic acid, somatostatin, vitamin K, multiple transfusions of RBC and fresh frozen plasma (FFP), and she underwent emergent upper GI endoscopy. The examination highlighted a foreign body immediately after the transition to the descending part of the duodenum. It was located in the posterior-inferior wall within the reticular crypt and adjacent to the foreign body. Bleeding was observed and treated with 12 cc of adrenaline infusion. The fragile clinical condition of the patient, combined with the recent use of anticoagulants, led the endoscopists to decide on the removal of the foreign body under general anesthesia in the operating room, accompanied by both gastroenterologists and general surgeons, in order to be prepared for any possible complication.

Furthermore, we conducted a new CT scan that compared to the previous one, the only different finding was a new small intestine obstruction. There was no alteration in the phenomenon of the inflammation and free air bubbles between the duodenal loop and the inside of the cecum pole. From the laboratory exams, an elevation of the inflammatory biomarkers and a drop in Hb is observed.

**Figure 2:**
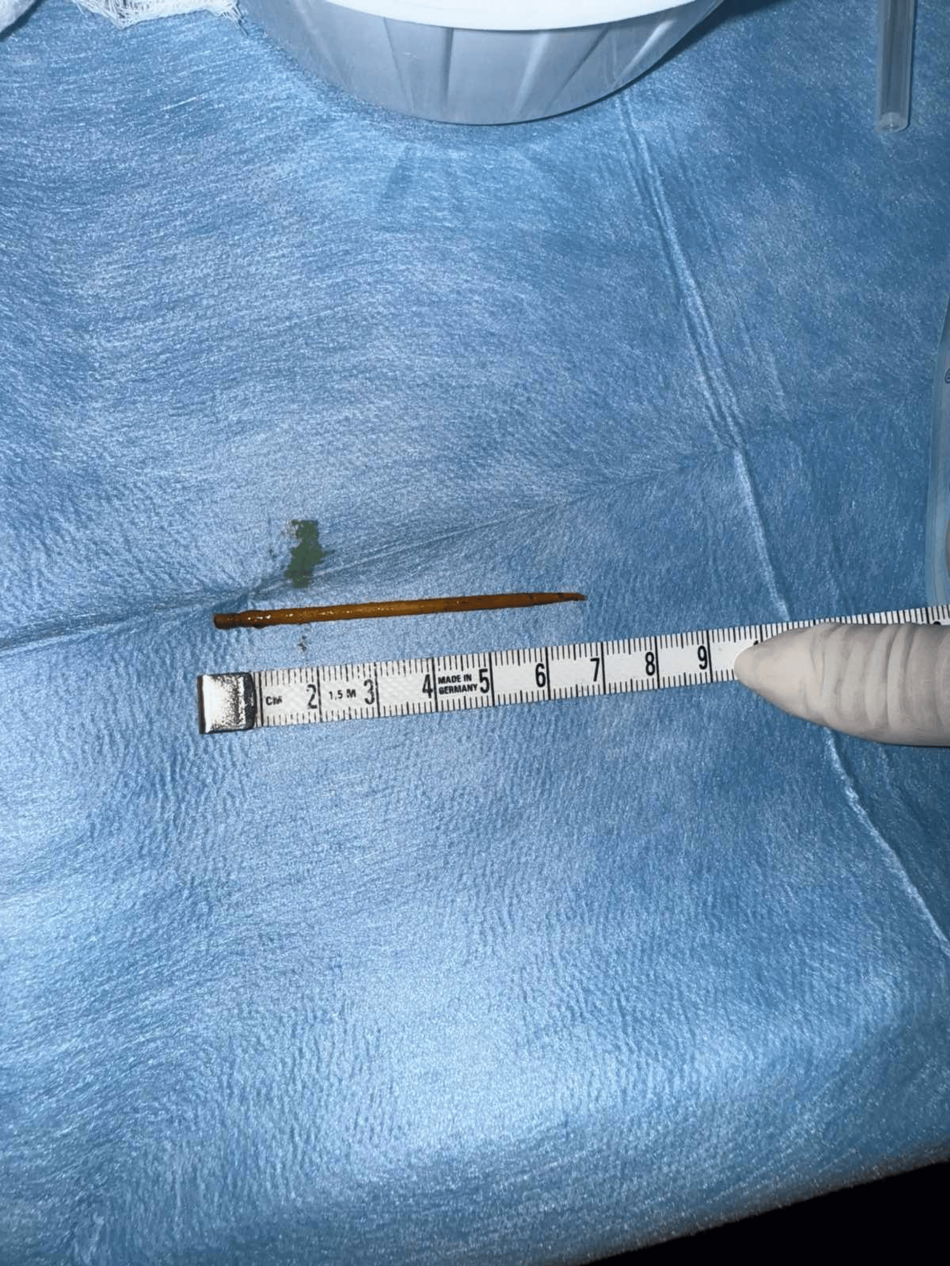
Foreign body - toothpick removed endoscopically.

The foreign body was removed endoscopically the following day. The foreign body was a 7 cm toothpick (Figure 2) that was removed with rat tooth surgical forceps from the second part of the duodenum, and afterward the defect was sealed using an over-the-scope clip (Ovesco Endoscopy, Tuebingen, Germany).

**Figure 3:**
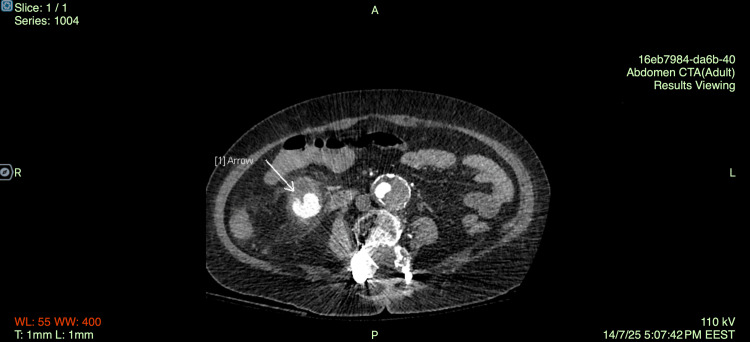
Axial CT angiography of the abdomen at the level of the infrarenal abdominal aorta demonstrates a fusiform aneurysmal dilatation with circumferential wall calcifications and probable mural thrombus.

**Figure 4:**
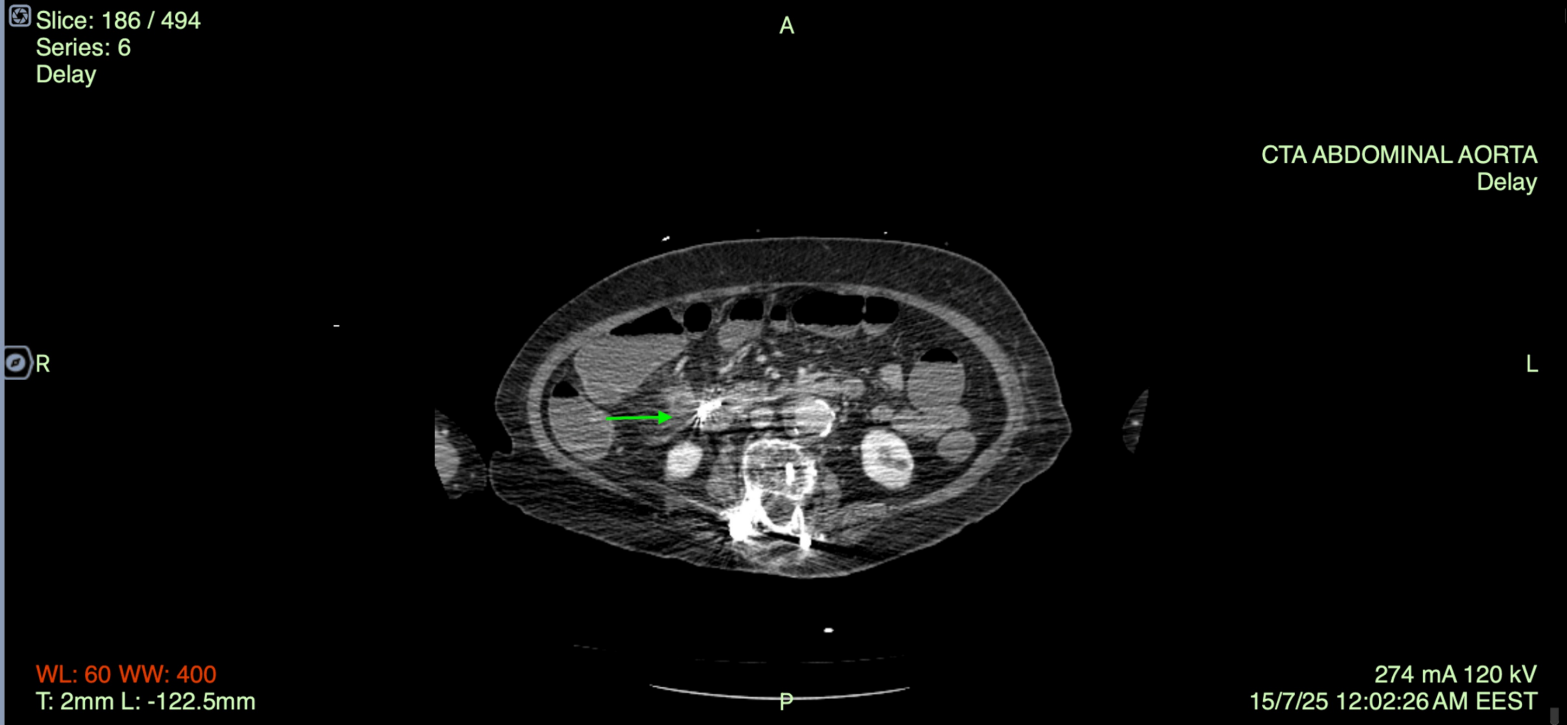
CT angiography - post-clipping.

Three days after the foreign body removal and while the patient showed clinical and laboratory improvement, she experienced three large-volume bowel movements containing blood clots and bright red blood. She was immediately transfused with RBC and FFP, a nasogastric tube was placed, and somatostatin and tranexamic acid were administered while concurrent hemodynamic monitoring was initiated. A new CT angiography was performed. On post-contrast imaging, an aneurysm of a branch of the SMA was identified (Figure 3), measuring approximately 9 mm in diameter, and there is evidence of active extravasation from the aneurysm. Furthermore, a subrenal abdominal aortic aneurysm with mural thrombus, measuring 4.6 cm, along with aneurysmal dilatation of the right common iliac artery was described. On the delayed phase images, contrast pooling was observed within the lumen of the cecum and ascending colon (Figure 4).

Communication between the inflammatory formation and the cecum was considered possible. After achieving hemodynamic stability with crystalloid solutions and RBC and FFP transfusions, the patient was transferred to Attikon University Hospital in order to undergo an arterial embolism performed by interventional radiologists. Indeed, a pseudoaneurysm of the ileocolic artery (branch of the upper mesenteric artery) was successfully embolized permanently with metallic coils.

After this embolization, the patient presented a rapid clinical and laboratory improvement with no other episodes of bleeding or pain. The inflammatory biomarkers were gradually reduced, and she gradually began internal nutrition without complications. She continued the antibiotic treatment. Seven days after the embolism, the patient underwent upper and lower GI endoscopy. The Ovesco clip was inspected without signs of bleeding, as well as this micronodule formation of mucous membranes, where biopsies were taken that were proved negative for malignancy. From the colonoscopy, polyps were observed throughout the entire length of the large intestine without recession and diverticulosis without inflammation (Table 2).

**Table 2:** Lab findings in the patient and the normal ranges.

Parameter	Patient’s value	Normal range
White blood cells (WBC)	16.91 × 10⁹/L (93.5% neutrophils)	4.0-10.0 × 10⁹/L
Hemoglobin (Hb)	11.6 g/dL	12.0-16.0 g/dL (female)
Hematocrit (Hct)	35.2%	36-46 % (female)
Platelets (PLT)	388 × 10⁹/L	150-400 × 10⁹/L
INR	1	0.8-1.2
Urea	32 mg/dL	15-40mg/dL
C-reactive protein (CRP)	118.8 mg/L	< 5 mg/L
Creatinine	0.7 mg/dL	0.6-1.1 mg/dL

## Discussion

Aneurysms of visceral arteries are extremely rare entities that affect only 0.2% [1] of the general population, and of these, approximately 5.5% involve the SMA [2]. Nevertheless, GI bleeding is the most common presentation and carries a high risk of mortality when rupture ensues [3,6]. Although they are usually silent and asymptomatic, they can present with abdominal pain and GI bleeding; therefore, they should be considered in the differential diagnosis when a patient presents with abdominal pain and anemia.

Visceral angiography is the gold standard method of diagnosing these aneurysms, because it allows us to understand the visceral anatomy and intervene if there is a reason. However, CT angiography, ultrasound and magnetic resonance angiography emerge the latest years and they offer a wide range of imaging that is close to 100% sensitivity [5,6] indeed, it has significant value before every treatment approach, the patient to undergo a CTA in order to specify the diameter and the location of the aneurysm, the amount of calcification, the possibility of a thrombus and the size of the feeding branches. This method is the least time-consuming and the most accurate in order to determine the treatment of choice [7].

Unlike true aneurysms, which require surgical treatment depending on their size (<25 mm, monitoring and conservative treatment are sufficient), pseudoaneurysms must always be treated surgically due to the increased risk of rupture. There are various therapeutic strategies that can be performed in these situations, which are categorized into open or laparoscopic surgical procedures and endovascular procedures [8].

It is widely acknowledged and recognized by the guidelines from The Society for Vascular Surgery that endovascular treatment is preferable to open surgery [9]. However, so far, there is no randomized controlled study or prospective study that compares the efficacy and safety of the available methods of treatment [10]. Endovascular therapies (EVTs) are gaining ground due to the shorter hospital stay, shorter operating time, and lower rates of cardiovascular complications [8]. These approaches usually include coils and micro-coils, plugs and covered stents, while liquid embolic agents and percutaneous injection of thrombin are also options [5].

The goal of EVT is to eliminate the aneurysm or the pseudoaneurysm from the arterial circulation. In this way, the bleeding can easily be controlled. This can be done by either blocking or preserving the flow through the pathological vessel, by coil embolization or stent placement, respectively [7]. The choice between preservation and occlusion depends on the location of perfusion as well as the collateral circulation [10]. Among the benefits of these techniques are that they can be performed under local anesthesia, and they are minimally invasive with early recovery [6], therefore, particularly suitable for patients with severe comorbidities or high surgical risk. However, there is a potential risk of end-organ ischemia due to occlusion of an artery and its collateral branches; thus, it is preferable to use techniques that preserve the artery [10].

Open or laparoscopic surgical repair continues to offer a safe and effective solution. The most important benefit of this technique is the possibility of a clear vision of the perforation and of the condition of the end-organ. The surgeon can easily recognize the ischemia and the need for revascularization during surgery. Furthermore, with open surgery, it is easier to isolate the aneurysm completely without damaging collateral circulation. However, nowadays surgery is performed only when EVT is difficult to perform or there are not adequate resources, because the perioperative mortality is higher during an open surgery than with endovascular approaches [5,10]. Available surgical techniques include aneurysmectomy with resection and end-to-end anastomosis, re-implantation, and simple ligation with or without arterial reconstruction [10,11]. In some cases, the surgeon may perform organ resection [10]. Proximal and distal ligation of the VAA without reconstruction is used in emergencies, such as frank rupture, or electively if collateral circulation is sufficient [6].

The aneurysms and pseudoaneurysms of SMA are very rare, as mentioned before, and they show high mortality rates after rupture [11]. They must be treated soon after they get diagnosed due to the high risk of rupture and complications [11]. Most cases are idiopathic or occur in the setting of infection, pancreatitis, or other inflammatory processes. To date and to our knowledge, there is only one case report of an aneurysm in SMA due to a swallowed foreign body [2]. In that setting, surgical management was performed; the pseudoaneurysm was opened, the wooden fragment was removed, and the artery was repaired with direct suture [12].

In our case, we chose a different strategy. The foreign body was removed endoscopically, followed by endovascular treatment to exclude the aneurysm. Although surgical intervention was the management of choice in the previously published report, the patient’s fragile clinical status and our review of similar case reports [13] supported a less invasive approach. In general, endovascular stent placement is considered the preferred treatment for mesenteric artery pseudoaneurysms, despite the inherent risk of end-organ ischemia [11].

## Conclusions

Pseudoaneurysm in the SMA is a rare but existing cause of upper abdominal bleeding that must be considered in every differential diagnosis. Acute abdominal pain and GI bleeding are the usual symptoms of a rupture of these aneurysms. There are two different treatment approaches, surgical and endovascular occlusion, with the latter gaining ground due to its lower complication rate and the reduction in both operative time and length of hospital stay. In this case, the patient received adequate conservative treatment and support for the acute, sharp abdominal pain and bleeding, followed by suitable imaging tests that led to the diagnosis of pseudoaneurysm and foreign body ingestion. The initial endoscopic removal of the foreign body with the placement of an Ovesco clip and subsequent embolization of the SMA led, despite the fragile clinical condition, to a normal postoperative course for the patient and complete recovery.
